# Family Anthropometric and Physiological Correlations With Parent‐ and Teacher‐Rated Preschooler Socio‐Behavioral Health

**DOI:** 10.1111/jcap.70056

**Published:** 2026-05-19

**Authors:** Jiying Ling, Ethan Yaroch, Yingcen Xie, Kenneth Resnicow, Jennifer Baumgartner

**Affiliations:** ^1^ Michigan State University College of Nursing East Lansing Michigan USA; ^2^ University of Minnesota School of Public Health Division of Epidemiology & Community Health Minneapolis Minnesota USA; ^3^ Clinical Research Branch, Division of Extramural Research, Basic and Mechanistic Research in Complementary and Integrative Health Branch Bethesda Maryland USA

**Keywords:** anthropometrics, early childhood, family demographics, physiological health, Socio‐behavioral development

## Abstract

**Purpose:**

This study investigated the correlations between family anthropometric and physiological health (i.e., body mass index [BMI], percent body fat, blood pressure [BP], skin carotenoids) and preschoolers' parent‐ and teacher‐rated socio‐behavioral outcomes (i.e., externalizing behaviors: self‐centered/explosive, attention problems, antisocial/aggressive behaviors; internalizing behaviors: social withdrawal, anxiety/somatic problems; social skills: social cooperation, interaction, and independence).

**Methods:**

Baseline data from a cluster randomized clinical trial were analyzed using R 4.0. A total of 168 preschoolers (*M*_*age*_ = 46.98 months) and 154 parents (*M*_*age*_ = 31.3 years) were non‐randomly recruited from 16 Head Start centers in the Midwest U.S. Both parents and teachers completed an online survey assessing preschoolers' socio‐behavioral health, and blinded trained data collectors measured each dyad's height, weight, percent body fat, skin carotenoids, and parents' BP. Generalized linear mixed‐effects models with restricted maximum likelihood estimation were applied, accounting for random cluster effects of childcare centers and classrooms.

**Findings:**

Preschoolers' BMI‐z was positively associated with teacher‐rated social skills (*B* = 3.66, *p* = 0.034). Parents' BMI had a positive relationship with preschoolers' self‐centered/explosive behaviors (*B* = 0.61, *p* = 0.031) and antisocial/aggressive behaviors (*B* = 0.50, *p* = 0.045), while their percent body fat and systolic BP were negatively associated with preschoolers' anxiety/somatic problems (*B* = −0.40, *p* = 0.034; *B* = −0.31, *p* = 0.032). Preschoolers' skin carotenoids were positively correlated with their social independence (*B* = 0.07, *p* = 0.025), and parents' skin carotenoids were negatively associated with preschoolers' antisocial/aggressive behaviors (*B* = −0.07, *p* = 0.038).

**Conclusions:**

The results offer important insights into the complex impact of poor anthropometric and physiological health on socio‐behavioral development during early childhood.

## 1 Introduction

Preschoolers' socio‐behavioral health is shaped by a range of biological factors, including obesity, diet, and parental health. Problem behaviors and low social skills are prevalent among preschoolers ages 3–5 years, significantly impacting their overall well‐being and future social adjustment (American Academy of Pediatrics 2025; Wichstrøm et al. 2012). Disruptive behaviors, defiance, and aggression are among the most frequently observed challenges in this age group, with approximately 15% of preschoolers exhibiting behavioral issues even in the absence of impairment (Bitsko et al. 2022; Rescorla et al. 2012). These behaviors are generally classified as either internalizing (e.g., anxiety, social withdrawal) or externalizing behaviors (e.g., aggression, hyperactivity) (Merrell 2002; Zhao et al. 2022). In addition to behavioral concerns, many preschoolers struggle with social competence, encompassing skills such as cooperation, communication, and emotional regulation (Martinsone et al. 2022; Huaqing Qi and Kaiser 2003). Early deficits in social competence have been linked to increased behavioral challenges in adolescence (Bornstein et al. 2010; Dong et al. 2023). Given this developmental trajectory, understanding the biological and environmental contributors to preschoolers' socio‐behavioral health is critical for early prevention efforts.

One key risk factor linked to preschoolers' negative socio‐behavioral health is obesity, because children with obesity are more likely to experience peer rejection, teasing, and social exclusion, which may lead to social withdrawal, emotional distress, and depressive symptoms (Sarrias and Blanco 2022; Zhang et al. 2024). However, only reliance on body mass index (BMI) may obscure these associations, as BMI is a limited indicator of adiposity and more precise measures, such as percent body fat, may provide clearer insight. Diet also plays a critical role in socio‐behavioral development, partially through the gut‐brain axis, with a diet rich in fruits and vegetables supporting gut microbiota diversity linked to improved cognitive and behavioral health (Flannery et al. 2020; Kraaij et al. 2023; Morse and Garcia 2025). Although skin carotenoids offer a non‐invasive biomarker of fruit and vegetable intake and have been explored in relation to cognitive and academic outcomes in adolescents (Cannavale et al. 2022; Rosok et al. 2025; Varghese et al. 2024), their associations with socio‐behavioral outcomes in preschoolers remain largely unexamined. In addition, parent (or legal guardian; thereafter referred to as “parent”) health factors, including weight status and blood pressure, may influence preschoolers' socio‐behavioral health through genetic and shared environmental pathways (Yeung et al. 2017; Zhang et al. 2022), yet most existing research has focused on adolescents rather than early childhood (Boyer and Nelson 2015; Maia et al. 2025). Together, these gaps highlight the need for research that examines more precise indicators of child adiposity (e.g., percent body fat), objective dietary biomarkers (e.g., skin carotenoids), and parent health factors in relation to socio‐behavioral outcomes during the preschool period.

To address these gaps in the literature, this cross‐sectional study examined the correlations between family anthropometric and physiological health indicators (i.e., BMI, percent body fat, skin carotenoids, and blood pressure) and preschoolers' socio‐behavioral outcomes, including problem behaviors and social skills. We hypothesized that better family anthropometric and physiological health would be associated with more favorable preschoolers' socio‐behavioral outcomes. Assessing preschoolers' socio‐behavioral functioning is challenging, particularly given the discrepancies between parent and teacher ratings. Parents generally report higher social skills and fewer behavioral concerns, whereas teachers—who observe children in structured peer environments—are more likely to identify peer‐related problem behaviors (Halliday et al. 2025; Thompson and Winsler 2018). These informant differences underscore the importance of incorporating multiple perspectives to obtain a comprehensive understanding of young children's socio‐behavioral health. Accordingly, we explored whether associations between family health indicators and preschoolers' socio‐behavioral outcomes differed by informant (parent *vs.* teacher), addressing the need for multi‐informant approaches in early childhood research.

### 1.1 Theoretical Framework

The study hypotheses were grounded in the Family Systems Theory, conceptualizing family as a dynamic system where each member's physiological health can shape the well‐being of the entire family, including early childhood development (Crossno 2011). When parents or children experience physiological health problems, parenting behaviors and parent‐child relationships can be harmfully affected (Brown and Brown 2015). Impaired parenting behaviors and strained parent‐child relationships can contribute to suboptimal child socio‐behavioral development, such as increased behavior problems (Cordts et al. 2020).

## 2 Methods

### 2.1 Design and Setting

The study is a secondary cross‐sectional analysis of baseline data collected as part of an ongoing cluster randomized control trial (RCT), which aimed to determine the effectiveness of a mindfulness‐based intervention on promoting preschoolers' mental, emotional, behavioral, and physical health (Ling et al. 2026). Baseline data were collected prior to randomization and intervention implementation. The parent trial randomizes clusters rather than individuals, and the current analysis focuses exclusively on pre‐intervention data. Parent‐preschool dyads were recruited from Head Start centers across Michigan. Head Start is a federally funded program of providing early childhood education to families under poverty or children in the foster care system to promote school readiness (Office of Head Start 2025). In the original trial, we included all siblings who met the eligibility criteria to avoid placing parents in the position of having to choose only one child to participate. To assess the potential impact of non‐independence, we conducted sensitivity analyses in which one child per family was randomly selected for inclusion. Results were consistent with analyses using the full dataset; therefore, all eligible children were retained in the final analyses.

### 2.2 Ethical Considerations

Both the original trial and the current study were approved by the Michigan State University Biomedical and Health Institutional Review Board (STUDY00009256 and STUDY202500783). In the original trial, parents provided consent and were formally informed that study data could be used for future research purposes. Thirty‐six teachers, each representing a different class (*n* = 36), provided informed consent to complete the survey assessing preschoolers' social and behavioral health. Preschoolers aged 5 years and older gave verbal assent prior to data collection.

All online survey data were collected and stored within a password‐protected Qualtrics account accessible only to authorized research team members. Data were subsequently downloaded and saved to the University's secure, access‐restricted study share drive for data cleaning and analysis. For in‐person data collection, paper forms were labeled with participant ID numbers only and did not contain identifying information. Completed forms were transported from childcare centers to the University in a locked box. After electronic data entry, the paper forms were stored in a locked file room within the college, accessible only to authorized research personnel.

### 2.3 Recruitment and Sample

Parents and their preschoolers aged 3–5 years old were recruited non‐randomly from 16 Head Start centers (7 urban, 9 rural) in Michigan during Fall 2024. Teachers distributed the study recruitment flyer containing a QR code and URL link for parents to access and complete the study's enrollment and screening survey. The study team also conducted in‐person visits to each center for recruitment and addressed any questions parents had.

Preschooler eligibility included enrollment in a Head Start program (full‐day or part‐day), being 3–5 years old at the start of the intervention, having no diagnosed health conditions restricting physical activity or fruit and vegetable consumption, and no diagnosed disorders causing severe communication and interaction difficulties. Parents were eligible if they provided consent, had at least weekly internet access, and were willing to participate in a mindfulness‐based intervention via Facebook or the study's website.

### 2.4 Data Collection

After confirming parent‐preschooler eligibility, parents received a text or email (depending on their contact preferences) with a link to the online baseline survey via Qualtrics. Parents had three business days to complete the survey before receiving a follow‐up reminder through email, text, or phone call. If the survey remained incomplete after seven business days, a third reminder was sent, and the classroom teacher was contacted to encourage completion. Additionally, the project manager provided weekly enrollment reports to Head Start classrooms to help teachers track enrollment progress in their classrooms. Teachers completed an online survey via Qualtrics to evaluate each enrolled preschooler's socio‐behavioral health.

Following the completion of online data collection, in‐person physical data collection was scheduled with each Head Start center. These sessions took place in private rooms within the participating centers, with teachers' presence. Trained data collectors measured parent‐preschooler dyads' height (cm), weight (kg), percent body fat, skin carotenoid levels (0–850), and parents' blood pressure (mmHg).

### 2.5 Measures

**Preschooler socio‐behavioral health** was assessed using the 76‐item Preschool and Kindergarten Behavior Scales‐Second Edition (PKBS‐2) (Merrell 2002). Each question used a 4‐point Likert format, with response options ranging from “never” to “often.” The PKBS‐2 has two subscales: social skills (34 items) and problem behaviors (42 items). The social skills subscale assesses social cooperation, interaction, and independence. The problem behaviors subscale evaluates externalizing behaviors (self‐centered/explosive, attention problems, antisocial/aggressive behaviors) and internalizing behaviors (social withdrawal, anxiety/somatic problems).

PKBS‐2 was standardized with a nationwide sample of 3317 children aged 3–6 years old (Merrell 1994). It demonstrates strong psychometric properties, with high internal consistency (Cronbach's *α* = 0.75–0.97) and moderate to high test‐retest reliability (*r* = 0.58–0.87). Its validity is supported by its ability to predict autism spectrum disorders (Edwards et al. 2003; Major et al. 2017; Merrell 1996). Raw subscale scores were converted to percentile ranks, with higher percentiles indicating stronger social competence on the social skills subscale and more pronounced behavioral problems on the problem behaviors subscale. In this study, the Cronbach's alpha coefficients were 0.92 and 0.97, 0.95 and 0.97, and 0.88 and 0.89 for parent‐ and teacher‐rated social skills (social cooperation: 0.88, 0.96; social interaction: 0.84, 0.93; social independence: 0.79, 0.91), externalizing problems (self‐centered/explosive: 0.90, 0.95; attention problems/overactive: 0.89, 0.95; antisocial/aggressive: 0.84, 0.89), and internalizing problems (social withdrawal: 0.78, 0.84; anxiety/somatic problems: 0.79, 0.81), respectively.

**Height, weight and percent body fat**. Height was measured to the nearest 0.1 cm using the ShorrBoard® Measuring Board Stadiometer. Weight and percent body fat were assessed using the InBody 270 body composition analyzer, a reliable and valid instrument for body composition analysis (Garcia et al. 2020; Larsen et al. 2021). This device has been successfully applied in studies involving preschoolers aged 3–5 years (Orsso et al. 2022; Sleiman et al. 2024). Weight was recorded to the nearest 0.1 kg, and percent body fat to the nearest 0.1%. To ensure measurement reliability, two measurements were taken for height, weight, and percent body fat. If the two measurements differed by ≥ 0.5 cm for height, ≥ 0.5 kg for weight, and ≥ 1% for percent body fat, a third measurement was taken by a different trained data collector. To minimize the impact of excessive food or drink intake, weight and percent body fat were measured before Head Start's scheduled breakfast or lunch.

During measurements, participants were asked to remove shoes, socks, bulky clothing, and hair accessories (when applicable). If the accessories could not be removed, trained data collectors used a flexible plastic ruler to measure hairdo height. The initial height measurement minus the height of the hairdo was used as the participant's height. After the height measurement, participants wiped their hands and feet with an InBody Tissue wipe before stepping onto the InBody platform and aligning their feet with the foot electrodes. Once the InBody system measured weight, a trained data collector entered each participant's study ID, biological sex, age, and average measured height (cm). When prompted, participants gripped the hand electrodes, ensuring correct thumb and finger placement. The trained data collectors then recorded percent body fat on the data collection form.

**Parent blood pressure** was measured to the nearest 1 mm Hg using the automatic SunTech CT40 device, following the guidelines from the Seventh Report of the Joint National Committee on Prevention, Detection, Evaluation, and Treatment of High Blood Pressure. The day before the appointment, parents were instructed to avoid smoking, exercise, and caffeine for at least 30 min prior to measurement; if these conditions were not met, the measurement was delayed accordingly. Blood pressure was taken using an appropriately sized cuff on the left arm (unless contraindicated) at three 30‐second intervals, and the readings were averaged. Afterward, parents were informed of their blood pressure results and provided an American Heart Association Blood Pressure Fact Sheet for further guidance.

**Skin carotenoids** for parent‐preschooler dyads were measured using the average of three consecutive scans taken at 5‐second intervals with a Veggie Meter® (Longevity Link Corporation). Carotenoids, a category of phytonutrients found in plants, are deposited in blood, skin, and other tissues, serving as a biomarker for fruit and vegetable intake (Aguilar et al. 2014; Radtke et al. 2021). The Veggie Meter® has been shown to be a reliable and non‐invasive tool for measuring skin carotenoids, including in preschool‐aged populations (May et al. 2020). For this study, children colored a picture to identify their dominant hand, and their non‐dominant index finger was cleaned with an alcohol wipe before measurement. Parents followed the same procedures for their non‐dominant ring finger.

**Family sociodemographic characteristics** were assessed using items adapted from the National Health Interview Survey. Parents reported their marital status, education level, employment status, annual family income, household child count, as well as their own and their preschooler's age, sex, ethnicity, and race when completing the enrollment and screening survey.

### 2.6 Data Analysis

Data analyses were conducted using IBM SPSS 28 and R 4.0. The “cdcanthro” R package was used to calculate preschoolers' BMI *z*‐score. Descriptive statistics (means, standard deviations, frequencies, percentages, and ranges) summarized demographic characteristics and outcomes. Pearson correlations examined the bivariate associations between anthropometric and physiological measures of both preschoolers and parents, and preschoolers' socio‐behavioral health percentile ranks rated by teachers and parents. To evaluate these relationships further, generalized linear mixed‐effects models with restricted maximum likelihood estimation were also applied, accounting for random cluster effects of childcare centers and classrooms. In addition, all family characteristics, including preschoolers' age, sex, ethnicity/race, parents' age, sex, ethnicity/race, marital status, employment status, education level, family annual income, and number of children, were adjusted. Categorical covariates were dichotomized: race (white vs. non‐white), marital status (married/partnered vs. separated/single), education level (≤ high school vs. > high school), and family annual income (<$20,000 vs. ≥$20,000). The Kenward‐Roger approximation method was adopted to estimate degrees of freedom. The results with a *p*‐value < 0.05 were considered as statistically significant. We did not adjust the significance level because our analyses focused on pre‐specified hypotheses grounded in a theoretical framework. Given the moderate sample size, applying adjustments could increase the risk of Type II errors, potentially obscuring meaningful associations (Rothman 2010).

**Post hoc sensitivity power analysis**. Using G*Power 3.1.9.7 (*F* tests; Linear multiple regression: Fixed model, *R* ^*2*^ increase), with *α* = 0.05 and power = 0.80, and assuming 2–8 tested predictors (13–19 total predictors), a sample size of 154–168 would be sufficient to detect effect sizes ranging from *f* ^*2*^ = 0.06 to 0.10 (small‐to‐medium effects).

## 3 Results

### 3.1 Demographic Characteristics

Among the 168 participating preschoolers, the mean age was 46.98 months (SD = 7.01, range = 34–63), with 49.4% being male. About 72% were White, 16.1% were multiracial, 8.9% were Black or African American, 1.2% were Asian, and 0.6% identified as other races. Additionally, 13.1% (*n* = 22) were Hispanic or Latino.

Among the 154 participating parents, the mean age was 31.3 years old (SD = 7.69, range = 19–69), with 5.2% being male. About 6.5% were Hispanic or Latino. The racial distribution was: 79.9% White, 9.1% Black or African American, 6.5% multiracial, 1.3% Asian, 0.6% Native Hawaiian or other Pacific Islander, and 0.6% other race. Nearly half of the parents (49.4%) were married or partnered, while 42.2% were single and 8.4% were separate, divorced, or widowed.

Regarding family income, 40.3% reported an annual family income of less than $20,000, 18.8% earned between $20,000 and $29,999, 22.7% reported between $30,000 and $49,999, and 18.2% had an annual family income of $50,000 or higher. Approximately 30.5% were employed full‐time, 27.9% worked part‐time, and 41.6% were unemployed. In terms of education level, 11% had less than a high school education, 35.4% were high school graduates, 33.8% completed some college, 11% graduated from a technical school or community college, and 7.7% had a bachelor's degree or higher. For weight status, 20.5% of the preschoolers were overweight, and 17.5% were obese. Among parents, 19.4% were overweight, and 58.1% were obese. Table 1 reveals the descriptions of the family anthropometric and physiological measures and preschoolers' behavioral health rated by parents and teachers.

**Table 1:** Descriptions of family anthropometric/physiological health and preschoolers' Socio‐Behavioral Outcomes.

Variable	Mean	SD
Anthropometric/physiological health		
Child percent body fat	24.24	6.87
Child BMI	17.01	3.09
Child BMI *z*‐score	0.74	1.35
Child BMI percentile	69.89	25.98
Child skin carotenoids	235.52	75.33
Parent percent body fat	40.51	11.99
Parent BMI	33.32	9.56
Parent skin carotenoids	229.48	81.27
Parent systolic blood pressure	120.63	15.35
Parent diastolic blood pressure	74.92	10.81
Parent rating		
Social skills	45.78	25.68
Social cooperation	38.47	24.62
Social interaction	56.18	26.99
Social independence	45.29	25.31
Problem behaviors	53.15	28.26
Externalizing problems	56.58	27.10
Self‐centered/explosive	60.38	26.54
Attention problems/overactive	60.75	27.77
Antisocial/aggressive	43.55	27.65
Internalizing problems	49.63	28.11
Social withdrawal	44.84	27.02
Anxiety/somatic problems	54.55	27.89
Teacher rating		
Social skills	43.67	32.10
Social cooperation	50.85	34.18
Social interaction	37.17	29.92
Social independence	44.86	29.98
Problem behaviors	38.48	29.56
Externalizing problems	39.25	31.88
Self‐centered/explosive	40.75	33.52
Attention problems/overactive	46.70	34.91
Antisocial/aggressive	30.68	28.16
Internalizing problems	38.61	28.75
Social withdrawal	49.59	31.76
Anxiety/somatic problems	35.34	28.39

### 3.2 Unadjusted Bivariate Associations

Table 2 shows the bivariate correlations between family anthropometric and physiological measures and preschoolers' socio‐behavioral health ratings by parents and teachers. Only preschoolers' BMI *z*‐score was positively related to their parent‐rated social interaction (*r* = 0.16, *p* = 0.045).

**Table 2:** Bivariate correlations between anthropometric/physiological health and preschoolers' socio‐behavioral outcomes.

Socio‐behavioral health	Child BMI‐*z*	Child Percent Body Fat	Child Skin Carotenoids	Parent BMI	Parent Percent Body Fat	Parent Skin Carotenoids	Parent Systolic BP	Parent Diastolic BP
Parent rating								
Social skills	0.11	−0.004	−0.02	−0.05	−0.11	−0.09	0.01	0.04
Social cooperation	0.06	−0.10	0.04	−0.05	−0.11	−0.04	−0.01	0.01
Social interaction	0.16*	0.14	−0.07	−0.09	−0.09	−0.10	−0.01	0.04
Social independence	0.17	−0.07	−0.03	0.04	−0.07	−0.15	0.05	0.06
Problem behaviors	−0.03	0.12	0.11	−0.03	−0.05	0.11	−0.02	0.03
Externalizing problems	−0.02	0.11	0.10	0.02	−0.02	0.10	0.01	0.07
Self‐centered/explosive	−0.06	0.13	0.08	0.03	0.01	0.06	0.05	0.11
Attention problems/overactive	0.01	0.11	0.09	0.02	−0.04	0.12	0.01	0.05
Antisocial/aggressive	0.04	0.06	0.13	0.004	−0.07	0.12	−0.03	0.03
Internalizing problems	−0.04	0.11	0.08	−0.09	−0.08	0.10	−0.05	−0.02
Social withdrawal	−0.04	0.09	0.11	−0.05	−0.10	0.07	−0.04	−0.03
Anxiety/somatic problems	−0.03	0.12	0.11	−0.11	−0.06	0.13	−0.04	0.01
Teacher rating								
Social skills	0.19*	0.05	0.17*	−0.17*	−0.08	0.09	−0.02	−0.01
Social cooperation	0.13	0.04	0.16*	−0.25**	−0.13	0.19*	−0.10	−0.10
Social interaction	0.20*	0.07	0.09	−0.14	−0.04	0.06	0.001	0.03
Social independence	0.19*	0.01	0.24**	−0.12	−0.06	0.06	−0.03	−0.02
Problem behaviors	−0.09	−0.06	−0.14	0.17*	0.02	−0.16	0.12	0.12
Externalizing problems	−0.05	−0.03	−0.13	0.24**	0.07	−0.22**	0.18*	0.16
Self‐centered/explosive	−0.05	−0.03	−0.15	0.25**	0.09	−0.23**	0.19*	0.16*
Attention problems/overactive	−0.08	−0.02	−0.13	0.22**	0.07	−0.18*	0.17*	0.14
Antisocial/aggressive	−0.03	−0.09	−0.05	0.22**	0.03	−0.17*	0.18*	0.16
Internalizing problems	−0.13	−0.08	−0.12	0.02	−0.06	−0.05	−0.003	0.03
Social withdrawal	−0.13	−0.05	−0.10	0.13	−0.01	−0.11	0.05	0.06
Anxiety/somatic problems	−0.09	−0.09	−0.12	−0.06	−0.09	−0.01	−0.03	0.01

* *p* < 0.05;

** *p* < 0.01.

For teacher‐rated social skills, preschoolers' BMI *z*‐score (*r* = 0.19, *p* = 0.016), skin carotenoids (*r* = 0.17, *p* = 0.032), and parents' BMI (*r* = −0.17, *p* = 0.038) were significantly associated. Specifically, preschoolers' BMI *z*‐score was positively related to their social interaction (*r* = 0.20, *p* = 0.010) and social independence (*r* = 0.19, *p* = 0.013). Preschoolers' skin carotenoids were positively correlated with their social cooperation (*r* = 0.16, *p* = 0.039) and social independence (*r* = 0.24, *p* = 0.003). Parents' BMI was negatively linked to preschoolers' social cooperation (*r* = −0.25, *p* = 0.002), while their skin carotenoids were positively related to preschoolers' social cooperation (*r* = 0.19, *p* = 0.019).

Regarding teacher‐rated externalizing problems, parents' BMI (*r* = 0.24, *p* = 0.002), skin carotenoids (*r* = −0.22, *p* = 0.008), and systolic BP (*r* = 0.18, *p* = 0.026) were significantly correlated. Particularly, parents' BMI was positively associated with preschoolers' self‐centered/explosive behaviors (*r* = 0.25, *p* = 0.001), attention problems/overactivity (*r* = 0.22, *p* = 0.006), and antisocial/aggressive behaviors (*r* = 0.22, *p* = 0.006). However, parents' skin carotenoids were negatively related to preschoolers' self‐centered/explosive behavior (*r* = −0.23, *p* = 0.006), attention problems/overactivity (*r* = −0.18, *p* = 0.026), and antisocial/aggressive behaviors (*r* = −0.17, *p* = 0.041). Parents' systolic BP was positively linked to preschoolers' self‐centered/explosive behaviors (*r* = 0.19, *p* = 0.021), attention problems/overactivity (*r* = 0.17, *p* = 0.166), and antisocial/aggressive behaviors (*r* = 0.18, *p* = 0.032). Moreover, their diastolic BP was positively correlated with preschoolers' self‐centered/explosive behaviors (*r* = 0.16, *p* = 0.048).

### 3.3 Adjusted Correlations for Parent‐Rated Preschoolers' Socio‐Behavioral Health

Tables 3 and 4 present the associations between family anthropometric and physiological measures and preschoolers' socio‐behavioral health rated by parents and teachers, while adjusting for family characteristics and cluster effects of childcare centers and classes. Among demographic covariates (see Supplementary Tables), preschoolers' age, female sex, and number of children were positively associated with social skills, indicating that older female preschoolers with more siblings exhibited greater social skills. Moreover, preschoolers whose parents were employed part‐time demonstrated lower social skills compared to those whose parents were employed full‐time. The number of children was negatively associated with externalizing problems, while an annual family income ≥ $20,000 showed a positive association. In addition, female preschoolers showed lower levels of internalizing problems, whereas parents' part‐time employment was positively associated with more internalizing problems.

**Table 3:** ^†^Relationship between preschooler anthropometric/physiological health and preschooler socio‐behavioral outcomes.

Socio‐behavioral health	BMI‐*z*			Percent Body Fat			Skin Carotenoids		
	*B*	95% CI	*p*‐value	*B*	95% CI	*p*‐value	*B*	95% CI	*p*‐value
Parent rating									
Social skills	1.54	−1.29, 4.37	0.303	0.07	−0.50, 0.64	0.813	−0.03	−0.09, 0.02	0.255
Social cooperation	0.60	−2.18, 3.38	0.683	−0.28	−0.85, 0.28	0.341	−0.02	−0.08, 0.04	0.484
Social interaction	2.99	0.01, 5.97	0.059	0.62	0.02, 1.22	0.050	−0.03	−0.09, 0.03	0.275
Social independence	0.13	−2.80, 3.05	0.934	−0.27	−0.87, 0.33	0.397	−0.02	−0.08, 0.03	0.426
Problem behaviors	−0.29	−3.66, 3.08	0.870	0.59	−0.13, 1.31	0.123	0.05	−0.02, 0.11	0.182
Externalizing problems	−0.25	−3.45, 2.96	0.885	0.42	−0.27, 1.10	0.251	0.06	−0.01, 0.12	0.095
Self‐centered/explosive	−0.99	−4.17, 2.20	0.559	0.38	−0.30, 1.06	0.294	0.05	−0.01, 0.11	0.139
Attention problems/overactive	0.59	−2.56, 3.75	0.723	0.51	−0.17, 1.18	0.158	0.06	0.00, 0.13	0.054
Antisocial/aggressive	0.69	−2.54, 3.92	0.689	0.24	−0.44, 0.93	0.498	0.05	−0.01, 0.11	0.143
Internalizing problems	−0.34	−3.69, 3.02	0.850	0.66	−0.05, 1.38	0.079	0.03	−0.03, 0.10	0.367
Social withdrawal	−0.22	−3.37, 2.93	0.896	0.63	−0.03, 1.30	0.072	0.03	−0.03, 0.09	0.386
Anxiety/somatic problems	−0.26	−3.63, 3.11	0.884	0.56	−0.15, 1.28	0.138	0.03	−0.04, 0.09	0.438
Teacher rating									
Social skills	3.66	0.37, 6.96	0.034*	0.15	−0.53, 0.82	0.678	0.03	−0.04, 0.09	0.409
Social cooperation	2.53	−1.15, 6.21	0.190	0.30	−0.47, 1.06	0.458	0.02	−0.05, 0.09	0.653
Social interaction	3.66	0.65, 6.68	0.021*	0.17	−0.45, 0.79	0.597	0.004	−0.05, 0.06	0.888
Social independence	3.74	0.58, 6.89	0.024*	0.003	−0.64, 0.65	0.993	0.07	0.01, 0.13	0.025*
Problem behaviors	−1.87	−4.91, 1.17	0.239	−0.14	−0.75, 0.47	0.653	−0.004	−0.06, 0.05	0.894
Externalizing problems	−1.05	−4.51, 2.42	0.564	−0.17	−0.89, 0.55	0.649	−0.01	−0.08, 0.06	0.798
Self‐centered/explosive	−1.42	−5.08, 2.23	0.457	−0.18	−0.94, 0.58	0.643	−0.02	−0.09, 0.05	0.656
Attention problems/overactive	−1.27	−5.04, 2.50	0.521	−0.20	−0.99, 0.58	0.623	−0.003	−0.08, 0.07	0.931
Antisocial/aggressive	−0.80	−3.96, 2.36	0.628	−0.30	−0.95, 0.35	0.376	0.01	−0.05, 0.07	0.827
Internalizing problems	−2.71	−5.63, 0.21	0.076	−0.15	−0.74, 0.44	0.630	−0.003	−0.06, 0.05	0.910
Social withdrawal	−3.52	−6.80, −0.25	0.040*	−0.04	−0.72, 0.64	0.908	0.002	−0.06, 0.07	0.958
Anxiety/somatic problems	−1.55	−4.39, 1.29	0.294	−0.19	−0.78, 0.40	0.528	−0.02	−0.07, 0.04	0.578

* *p* < 0.05.

^†^ Adjusted for family characteristics and cluster effects of center and class; BMI: body mass index; B: regression coefficient; 95% CI: 95% confidence interval for B.

**Table 4:** ^†^relationship between parent anthropometric/physiological measures and preschooler socio‐behavioral health.

Socio‐behavioral health	BMI			Percent Body Fat			Skin Carotenoids			Systolic BP			Diastolic BP		
	*B*	95% CI	*p*	*B*	95% CI	*p*	*B*	95% CI	*p*	*B*	95% CI	*p*	*B*	95% CI	*p*
Parent rating															
Social skills	−0.10	−0.53, 0.34	0.674	−0.11	−0.48, 0.26	0.566	−0.03	−0.08, 0.03	0.384	0.04	−0.24, 0.31	0.806	0.08	−0.29, 0.46	0.682
Social cooperation	−0.12	−0.55, 0.31	0.593	−0.10	−0.46, 0.27	0.605	−0.02	−0.08, 0.04	0.479	−0.03	−0.29, 0.24	0.850	−0.02	−0.39, 0.35	0.926
Social interaction	−0.20	−0.67, 0.26	0.409	−0.11	−0.51, 0.28	0.589	−0.01	−0.07, 0.05	0.691	0.02	−0.27, 0.31	0.882	0.05	−0.36, 0.45	0.825
Social independence	0.13	−0.31, 0.57	0.570	−0.03	−0.40, 0.35	0.895	−0.05	−0.10, 0.01	0.124	0.11	−0.16, 0.38	0.454	0.18	−0.19, 0.55	0.364
Problem behaviors	−0.08	−0.60, 0.43	0.762	−0.09	−0.54, 0.36	0.708	0.02	−0.05, 0.09	0.564	−0.14	−0.46, 0.19	0.432	0.05	−0.40, 0.49	0.850
Externalizing problems	0.05	−0.44, 0.54	0.849	−0.01	−0.44, 0.42	0.949	0.02	−0.04, 0.09	0.479	−0.06	−0.37, 0.25	0.698	0.15	−0.28, 0.57	0.518
Self‐centered/explosive	0.07	−0.42, 0.56	0.776	−0.001	−0.43, 0.42	0.995	0.02	−0.05, 0.08	0.606	−0.03	−0.34, 0.28	0.853	0.19	−0.23, 0.61	0.400
Attention problems/overactive	0.01	−0.47, 0.50	0.956	−0.07	−0.50, 0.35	0.738	0.04	−0.02, 0.10	0.251	−0.03	−0.34, 0.27	0.831	0.14	−0.28, 0.56	0.548
Antisocial/aggressive	0.09	−0.40, 0.59	0.727	0.03	−0.40, 0.59	0.885	0.02	−0.05, 0.08	0.657	−0.12	−0.43, 0.19	0.466	0.06	−0.36, 0.49	0.776
Internalizing problems	−0.23	−0.74, 0.28	0.399	−0.15	−0.60, 0.29	0.502	0.02	−0.05, 0.08	0.676	−0.21	−0.53, 0.11	0.223	−0.11	−0.55, 0.33	0.653
Social withdrawal	−0.08	−0.56, 0.40	0.760	−0.19	−0.61, 0.22	0.367	0.01	−0.06, 0.07	0.854	−0.16	−0.46, 0.14	0.328	−0.09	−0.51, 0.32	0.668
Anxiety/somatic problems	−0.29	−0.81, 0.22	0.275	−0.08	−0.52, 0.37	0.746	0.03	−0.04, 0.09	0.486	−0.19	−0.51, 0.13	0.276	−0.04	−0.48, 0.41	0.879
Teacher rating															
Social skills	−0.03	−0.53, 0.47	0.902	0.27	−0.15, 0.70	0.216	−0.01	−0.07, 0.06	0.866	0.09	−0.22, 0.40	0.578	0.06	−0.38, 0.49	0.799
Social cooperation	−0.37	−0.93, 0.18	0.200	0.12	−0.36, 0.60	0.641	0.04	−0.03, 0.11	0.332	−0.08	−0.42, 0.27	0.666	−0.17	−0.64, 0.31	0.511
Social interaction	−0.02	−0.48, 0.44	0.946	0.29	−0.09, 0.66	0.144	−0.004	−0.06, 0.06	0.897	0.08	−0.21, 0.37	0.584	0.07	−0.34, 0.47	0.748
Social independence	0.08	−0.40, 0.56	0.747	0.21	−0.20, 0.62	0.317	−0.01	−0.07, 0.05	0.759	0.07	−0.24, 0.37	0.679	0.09	−0.34, 0.51	0.692
Problem behaviors	0.19	−0.28, 0.65	0.440	−0.27	−0.66, 0.12	0.181	−0.03	−0.09, 0.03	0.425	0.06	−0.23, 0.35	0.677	0.12	−0.29, 0.52	0.575
Externalizing problems	0.52	0.01, 1.04	0.055	−0.09	−0.55, 0.36	0.692	−0.07	−0.14, 0.00	0.059	0.28	−0.04, 0.61	0.101	0.34	−0.12, 0.79	0.162
Self‐centered/explosive	0.61	0.07, 1.15	0.031*	−0.01	−0.48, 0.47	0.982	−0.07	−0.14, 0.00	0.059	0.29	−0.05, 0.63	0.107	0.31	−0.17, 0.78	0.219
Attention problems/overactive	0.45	−0.12, 1.03	0.134	−0.18	−0.13, 0.02	0.500	−0.05	−0.13, 0.02	0.191	0.30	−0.06, 0.66	0.113	0.36	−0.14, 0.85	0.176
Antisocial/aggressive	0.50	0.03, 0.98	0.045*	−0.04	−0.45, 0.38	0.869	−0.07	−0.13, −0.01	0.038*	0.27	−0.04, 0.58	0.104	0.38	−0.05, 0.81	0.096
Internalizing problems	−0.27	−0.72, 0.19	0.256	−0.40	−0.77, −0.03	0.039*	0.04	−0.02, 0.10	0.213	−0.23	−0.51, 0.06	0.128	−0.17	−0.57, 0.23	0.420
Social withdrawal	0.07	−0.44, 0.58	0.797	−0.27	−0.70, 0.16	0.226	0.003	−0.06, 0.07	0.927	−0.04	−0.36, 0.28	0.807	0.04	−0.41, 0.48	0.877
Anxiety/somatic problems	−0.41	−0.84, 0.03	0.071	−0.40	−0.76, −0.04	0.034*	0.05	0.00, 0.11	0.075	−0.31	−0.59, −0.03	0.032*	−0.28	−0.68, 0.11	0.169

* *p* < 0.05.

^†^ Adjusted for family characteristics and cluster effects of center and class; BMI: body mass index; BP: blood pressure; B: regression coefficient; 95%CI: 95% confidence interval for B.

### 3.4 Adjusted Correlations for Teacher‐Rated Preschoolers' Socio‐Behavioral Health

Tables 3 and 4 showed that preschoolers' BMI‐z was positively associated with overall social skills (*B* = 3.66, *p* = 0.034), social interaction (*B* = 3.66, *p* = 0.021) and social independence (*B* = 3.74, *p* = 0.024), but negatively related to social withdrawal (*B* = −3.52, *p* = 0.040). Additionally, preschoolers' skin carotenoids were positively correlated with their social independence (*B* = 0.07, *p* = 0.025). Regarding parents' anthropometric and physiological measures, parents' BMI had a positive relationship with preschoolers' self‐centered/explosive behaviors (*B* = 0.61, *p* = 0.031) and antisocial/aggressive behaviors (*B* = 0.50, *p* = 0.045). Parents' percent body fat was negatively associated with preschoolers' internalizing problems (*B* = −0.40, *p* = 0.039), particularly anxiety/somatic problems (*B* = −0.40, *p* = 0.034). Moreover, parents' skin carotenoids were negatively associated with preschoolers' antisocial/aggressive behaviors (*B* = −0.07, *p* = 0.038), and parents' systolic BP was negatively related to preschoolers' anxiety/somatic problems (*B* = −0.31, *p* = 0.032).

As demonstrated in the supplemental tables, older female preschoolers showed greater social skills. For teacher‐rated externalizing problems, both the number of children and parent education beyond high school were negatively associated with this outcome, whereas having a female parent was positively correlated with externalizing problems. Regarding internalizing problems, female preschoolers exhibited lower levels of internalizing problems, while non‐Hispanic parents were positively associated with higher internalizing problem scores.

## 4 Discussion

This study is among the first to examine the relationships between family anthropometric and physiological health and preschoolers' socio‐behavioral outcomes using both parent and teacher reports. Notably, preschoolers' BMI‐z was positively associated with teacher‐rated social skills, and parents' anthropometric and physiological indicators were primarily correlated with teacher‐reported problem behaviors. In addition, higher skin carotenoids in both preschoolers and parents were linked to more favorable preschooler socio‐behavioral outcomes. Several family demographic factors, including child age and sex, parent employment and education, and household size, were also associated with preschoolers' socio‐behavioral health.

Contrary to prior research linking body weight with stigma, peer rejection, and social difficulties in older children (Cheng et al. 2022; Pont et al. 2017), this study did not find evidence that preschoolers' excess body weight was detrimental to their social skills. This discrepancy may reflect developmental differences, as peer relationships among preschoolers are primarily characterized by acceptance rather than the rejection or victimization commonly observed in school‐age children and adolescents (Bierman et al. 2010; Burke et al. 2023). Thus, excess body weight may not yet contribute to negative social experiences during early childhood. Differences in sample characteristics among studies, including daycare enrollment, socioeconomic status, and family dynamics, may also contribute to the inconsistent results (Cawley and Spiess 2008). Hence, further investigation is warranted to explore the potential mechanisms linking anthropometrics to socio‐behavioral health. Biologically, excess adiposity may contribute to increased systematic low‐grade inflammation and alterations in the gut‐brain axis, which can influence neural circuits involved in emotional processing and behavioral regulation (Leigh and Morris 2020). Psychosocially, body composition may influence children's socio‐emotional development through mechanisms such as self‐regulation and resilience (Evans et al. 2012; Yang et al. 2022). Environmentally, broader contextual factors including family dynamics and social settings, may interact with parental health to influence children's social‐behavioral development (Pierce et al. 2022). These pathways likely operate in combination, highlighting the need for integrative research examining biological, psychosocial, and environmental mechanisms simultaneously.

Unlike preschoolers' own body composition, higher parent BMI was associated with increased teacher‐reported externalizing behaviors, particularly self‐centered/explosive and antisocial/aggressive behaviors. These results are somewhat consistent with the literature showing that maternal obesity is associated with externalizing behaviors in children, especially when assessed by teachers (Mikkelsen et al. 2017; Parker et al. 2022). One possible mechanism is the altered offspring neurodevelopment, as maternal obesity has been associated with reduced functioning within prefrontal and limbic networks involved in emotional and behavioral regulation (Parsaei et al. 2024). Disruptions in these neural systems may impair children's ability to regulate emotions and behaviors effectively. Consequently, higher parental BMI may contribute to poorer emotional and behavioral regulation in offspring, which in turn may increase the likelihood of externalizing behaviors (Godfrey et al. 2017). Another likely explanation is as parents with obesity may engage in more controlling and reduced responsive parenting practices, potentially undermining children's emotion regulation and increasing behavior problems (Hernandez Acton et al. 2024; Wang et al. 2022). Together, these findings highlight the importance of family‐centered approaches to foster optimal early childhood development.

Interestingly, parents' percent body fat and systolic blood pressure were associated with lower levels of teacher‐rated internalizing behaviors, contrasting with prior research (Parker et al. 2022). Although human evidence is limited, emerging animal studies suggest that the behavioral impact of parental obesity may depend on dietary composition. For example, experimental studies in zebrafish suggest that offspring exposed to parental high‐fat diets exhibit greater anxiety‐like behaviors compared with those exposed to high‐carbohydrate diets (Türkoğlu et al. 2022). These effects may reflect diet‐dependent metabolic programming involving triglyceride metabolism, differential activation of the Peroxisome Proliferator‐Activated Receptor (PPAR) gene family regulating appetite‐related genes, and oxidative stress‐related neuronal damage (Türkoğlu et al. 2022). Due to the low‐income context of the current sample, dietary patterns characterized by higher intake of unhealthy fats may partially explain the results (Khor and Ooi 2025). Future research should examine how specific dietary patterns interact with anthropometric health to influence children's mental well‐being. Given the very limited research examining intergenerational links between parental cardiometabolic health and child mental health, these findings should be interpreted cautiously and warrant further investigation using longitudinal designs in larger and more diverse samples.

This study also provides preliminary evidence supporting the beneficial effects of family skin carotenoids on preschoolers' socio‐behavioral development. Skin carotenoids, a biomarker of fruit and vegetable intake, have been linked to reduced stress, anxiety, and depression in adults (Rasmus and Kozłowska 2023), as well as improved cognitive development in toddlers (Rosok et al. 2024) and academic achievements in school‐age children (Rosok et al. 2025). Consistent with prior work, greater consumption of fruits and vegetables has been associated with better emotion regulation (Santos et al. 2022) and fewer behavioral problems in preschoolers (Choi et al. 2018). These findings suggest that interventions promoting fruit and vegetable intake at the family level may support young children's socio‐behavioral health.

Consistent with existing literature (Gomes and Pereira 2014), older female preschoolers demonstrated higher social skills and fewer internalizing problems compared to their peers. This may be due to gender socialization development in early childhood, as girls are more likely than boys to accept same‐sex peers with low social acceptance (Sebanc et al. 2003). Sibling dynamics also appeared influential, as having more siblings was associated with lower externalizing behaviors, although prior research suggests more complex patterns depending on sibling type and family structure (Fomby et al. 2016). These mixed results indicate the need to further explore how family composition shapes early socio‐behavioral development.

Finally, children of parents employed part‐time exhibited more socio‐behavioral difficulties than those with full‐time employed parents. Prior research found that part‐time employed mothers often assumed more household and childcare responsibilities, which might increase family stress and strain marital quality (Buehler et al. 2011; Sztányi‐Szekér et al. 2024). However, evidence on part‐time employment and family well‐being is mixed (Buehler and O'Brien 2011), underscoring the importance of considering employment status alongside broader sociodemographic contexts, including education and socioeconomic resources, when designing culturally responsive interventions.

## 5 Limitations

This study has a few limitations. The cross‐sectional nature of the study data restricts from making any causal inferences, and it is highly plausible that poor socio‐behavioral health may play a role in the development of obesity and physiological health problems (Derks et al. 2019). The proxy‐reported data on preschoolers' socio‐behavioral health may be subject to social desirability and recall bias. However, the inclusion of objectively measured anthropometric and physiological health markers enhances the validity of the findings. The generalizability of the results is limited to preschoolers and families from low socioeconomic backgrounds.

## 6 Conclusions

The study provides insight into the relationships between family anthropometric and physiological health and preschoolers' socio‐behavioral outcomes. Particularly, preschoolers' teacher‐rated social skills were positively associated with their BMI *z*‐score, while parents' high BMI was related to greater teacher‐reported externalizing behaviors. These results highlight the complexity of family health dynamics and underscore the need to further investigate biological, psychosocial, and environmental mechanisms that may contribute to preschoolers' socio‐behavioral development. Additionally, the beneficial effects of skin carotenoids suggest that family‐based interventions promoting fruit and vegetable intake may support preschoolers' socio‐behavioral health. When designing culturally responsive interventions, family sociodemographic factors, including child age and sex, parent employment and education, and sibling dynamics, should be carefully considered. Future longitudinal research is needed to clarify causal pathways linking family health indicators with preschoolers' social skills and problem behaviors.

## Author Contributions

**Jiying Ling:** conceptualization, supervision, funding acquisition, investigation, project administration, methodology, writing – original draft. **Ethan Yaroch:** data curation, project administration, writing – original draft. **Yingcen Xie:** data curation, formal analysis, project administration, visualization, writing – original draft. **Kenneth Resnicow:** conceptualization, investigation, methodology, writing – review and editing. **Jennifer Baumgartner:** methodology, writing – review and editing.

## Conflicts of Interest

The authors declare no conflicts of interest.

## Supporting information


**Table S1**: Associations between family demographic characteristics and preschoolers' caregiver‐rated socio‐behavioral health. **Table S2**: Associations between family demographic characteristics and preschoolers' teacher‐rated socio‐behavioral health.

## Data Availability

The data that support the findings of this study are available on request from the corresponding author. The data are not publicly available due to privacy or ethical restrictions.
